# Biomechanical analysis of lower limbs during stand-to-sit tasks in patients with early-stage knee osteoarthritis

**DOI:** 10.3389/fbioe.2023.1330082

**Published:** 2023-12-20

**Authors:** Jing Pan, Wenqin Huang, Zhiguan Huang, Jun Luan, Xiaohui Zhang, Bagen Liao

**Affiliations:** ^1^ Department of Sports Medicine, Guangzhou Sport University, Guangzhou, China; ^2^ School of Sports and Health, Guangzhou Sport University, Guangzhou, China; ^3^ Guangzhou Eleventh People’s Hospital, Guangzhou, China

**Keywords:** osteoarthritis, knee osteoarthritis (KOA), early-stage, stand-to-sit task, biomechanics, sEMG

## Abstract

**Background:** Knee osteoarthritis (KOA) is a common degenerative disease among the older people that severely affects their daily life. Previous studies have confirmed that movement biomechanics are altered in patients with KOA during task performance. However, changes that occur in lower limb joints and muscles in the three planes during stand-to-sit (STS) tasks in patients with early-stage KOA are unclear.

**Method:** Of the 36 participants recruited in this study, 24 (8 males and 16 females) and 12 (4 males and 8 females) were added to the KOA and control groups, respectively. The Nexus Vicon motion capture system along with Delsys wireless surface electromyography devices and plantar pressure measurement mat was used to record test data. A Visual 3D software was used to process the data and calculate the biomechanical and electromyographic parameters during STS tasks.

**Results:** There was no significant difference in task duration between the two groups. Patients with KOA could perform a greater range of pelvic motion and smaller range of hip and knee joint motion with a lower maximum hip joint angular acceleration in the sagittal plane and greater knee and ankle joint motion in the coronal plane. There was no significant difference in the motion range in the horizontal plane. During the STS task, patients in the KOA group had a lower vertical ground reaction force (GRF) amplitude on the injured side but a higher integrated GRF on both sides than those in the control group. Moreover, patients with KOA demonstrated higher PERM and PABM of the lower limb joints and smaller knee PADM and ankle PEM. Additionally, maximum activation levels of GMed muscle, affected-side gluteus medius (GM), ST, rectus femoris (RF), and tibialis anterior (TA) muscles were lower in patients with KOA than in controls. Conversely, the activation level of biceps femoris (BF) was higher. Furthermore, the integral EMG values of GMed, GM, ST, VL, RF, vastus medialis VM, and TA muscles on the affected side were lower, except for the BF muscle, in patients with KOA.

**Conclusion:** Compared with the participants in the control group, patients with early-stage KOA exhibited consistent changes in sEMG parameters and biomechanical alterations in the sagittal plane, as observed in previous studies. However, differences in parameters were observed in the coronal and transverse planes of these patients. The noninvasive analysis of the 3D parameters of the involved motion patterns may lead to the early detection of KOA.

## 1 Introduction

Knee osteoarthritis (KOA) is a prevalent disease that primarily manifests as chronic pain around the joints (Allen and Golightly, 2015), which is emerging as a leading cause of functional disability in adults (Glyn-Jones et al., 2015). KOA is particularly common among individuals aged >60 years, with global prevalence rates of 20% and 10% among women and men, respectively (Stubbs et al., 2016). Considering the aging population and escalating obesity rates, the incidence of KOA is predicted to considerably increase and impose a substantial economic burden on society (Litwic et al., 2013).

KOA is a complex disease, with its pathogenesis potentially encompassing various fields such as biomechanics and biochemistry. The mechanism of the onset and progression of KOA is unclear, and the treatment options are limited (Glyn-Jones et al., 2015). To date, no drugs have been identified that can effectively delay or prevent the structural deterioration associated with KOA (Zhu et al., 2018). Consequently, the discovery of methods for the early identification and prevention of KOA is a pressing global concern.

The evaluation of sit-to-stand and stand-to-sit (STS) postural transitions serves multiple purposes (Nevitt et al., 1989; Lord et al., 2002). As individuals age, these transitions become increasingly challenging daily tasks (Galán-Mercant and Cuesta-Vargas, 2013). Some studies have reported changes in the neuromuscular activation and movement patterns while performing daily activities in patients with KOA (Man and Mologhianu, 2014; Ghazwan et al., 2022), which can significantly impact their quality of life (Bijlsma et al., 2011).

The biomechanical characteristics of STS tasks in patients with KOA have been extensively examined (Bouchouras et al., 2015; Sonoo et al., 2019). However, there is limited research on the STS task performed by patients with KOA with a primary focus on biomechanical changes in the sagittal plane; therefore, a comprehensive evaluation of changes in all three planes has not yet been made (Wu et al., 2015; Wilk et al., 2018; Fu et al., 2021). Furthermore, an in-depth exploration of notable characteristics of early-stage patients is warranted. A comprehensive study of patients with early-stage KOA will help in enhancing our understanding of the onset and progression of KOA as well as enabling its early diagnosis.

Therefore, this study aimed to observe alterations in pelvic and lower extremity biomechanics and surface electromyography (sEMG) data in all three planes in patients with early-stage KOA during STS task. These findings may provide biomechanical insights that might facilitate the early identification and development of prevention strategies for KOA.

## 2 Materials and methods

### 2.1 Subjects and measurement protocol

The Human Subject Committee of Guangzhou Sport University (2022LCLL-32) approved the protocol for this case-control study. Before participation, all participants were provided with detailed information regarding the study, and they provided informed consent for participation in this study.

Two groups of participants were recruited for this study. Participants who were diagnosed with unilateral/bilateral mild-moderate KOA (Ribeiro et al., 2020) by a doctor using anterior X-ray images were recruited to the first group. The inclusion criteria for patients with KOA were as follows: aged 55–75 years and had the Kellgren–Lawrence (K–L) grade of I or II. We recruited sex- and age-matched healthy individuals in the control group. Participants were excluded if they had the following symptoms: hip or ankle joint injuries, knee joint pain caused by other reasons, obvious low back pain (VAS ≥3′) (Williamson and Hoggart, 2005), recent cerebrovascular accident or myocardial infarction, steroid injection within 6 months, fractures, Parkinson’s and other diseases due to which they could not complete STS task. Finally, 36 participants were included in the trial, of whom 24 (8 males and 16 females) and 12 (4 males and 8 females) were added to the KOA and CG group, respectively.

**FIGURE 1:**
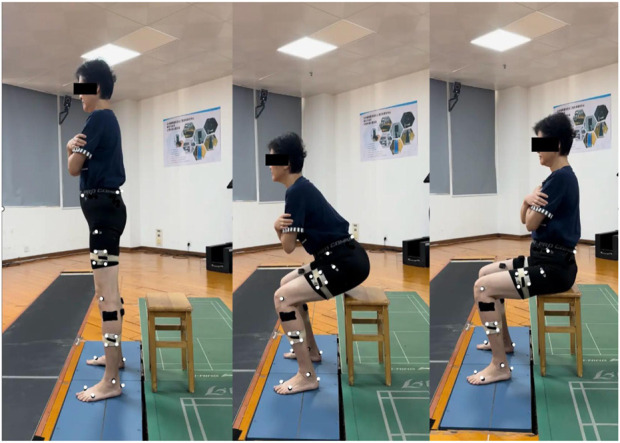
Stand-to-sit test.

The 30-s chair standing test (30sCST) is one of the five physical function tests recommended by the International Society for the Study of Osteoarthritis for patients with KOA (Dobson et al., 2013). Impaired postural balance and biomechanical changes can be more easily captured when STS task is performed repeatedly than when it is performed only once (Fu et al., 2021). According to the procedure mentioned in OARS I (Dobson et al., 2013), a chair without a backrest or armrest with a height of 43 cm (17 inches) was used for the measurement (Figure 1). Before the experiment, participants were asked to sit on the chair, cross their arms, and place their upper arms close to the chest to avoid obstructing lower limb marks. The position of their was unrestricted. At the beginning of the task, the rose at a natural speed, then sat down, and repeated this movement within 30 s. Each participant were given three trials and was thoroughly familiarized with the process before the formal trial began.

### 2.2 Multiparametric characterization of STS movement

For the analysis, the injured side or the side with a more serious imaging diagnosis was noted in patients with unilateral/bilateral symptoms, and the dominant side was noted in the control group. The dominant leg was determined by asking three standard questions: which leg would you use to 1) play football, 2) step on a worm, and 3) draw a diamond on the ground (Vauhnik et al., 2008)?

#### 2.2.1 Temporal parameters

The temporal parameters explored in this study included STS time and T1 time (%). The start of the task was defined as the first transition of the hip flexion angular velocity from negative to positive. The end of the task was defined as the moment when the vertical ground reaction force is ≤ 10N. T1 was defined as the time of maximum hip flexion angle during sitting phase (Galli et al., 2008; Norman-Gerum and McPhee, 2020).

#### 2.2.2 Kinematic and kinetic parameters

**FIGURE 2:**
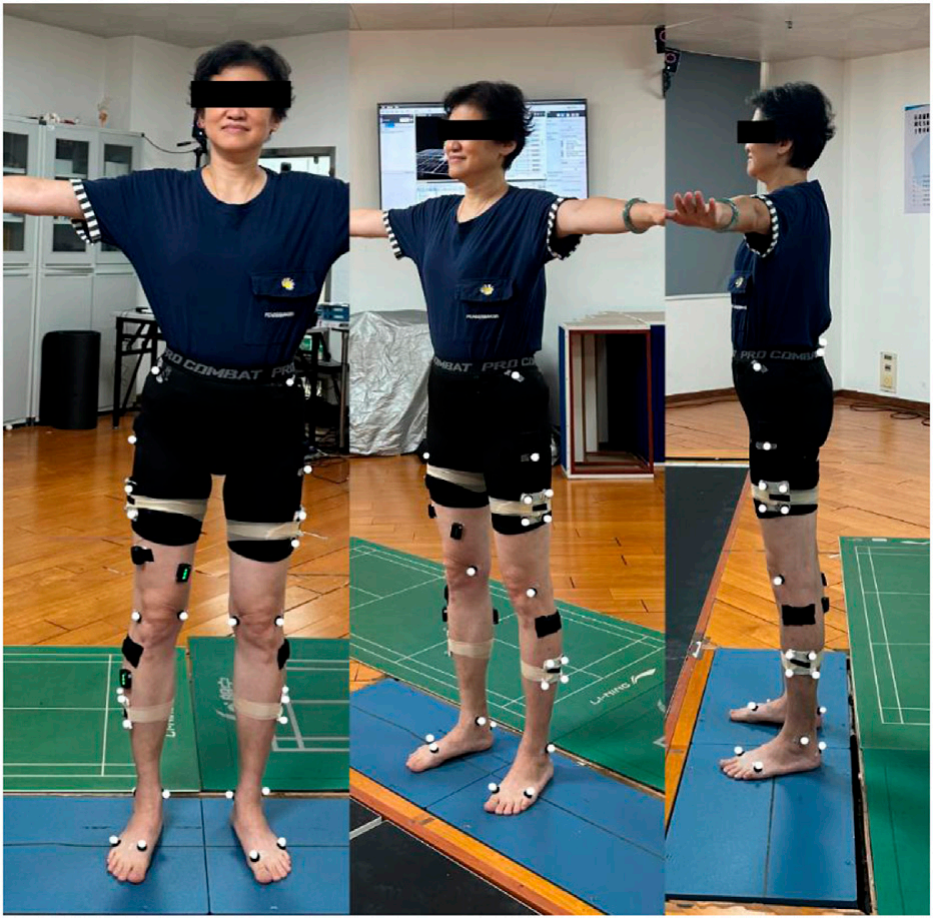
Retroreflective markers placement.

The methods for collecting data regarding the 3D kinematic and kinetic parameters of the participant were as follows: the kinetic raw data included joint moment and vertical ground reaction forces (vGRF), which were normalized for the participants’ weight; the kinematic raw data included joint velocity and ROM. Two force platforms (AMTI OR6-7, Watertown, MA, United States, 60 × 40 cm) were used to record the kinetic parameters of the lower body at 1,000 Hz during the task. The trajectories of the lower body were recorded using a motion capture system (Nexus Vicon, Oxford, United Kingdom) equipped with 10 infrared induction cameras and sampled at 100 Hz. The Nexus Vicon system relies on infrared sensors to capture motion and requires sufficient retroreflective markers to reflect the location of the limb in space. The positions of the markers are shown in Figure 2 and their names are shown in Table 1.

**TABLE 1:** 28 retroreflective markers placement.

Marker name	Marker location
L/R_IAS	Anterior superior iliac spine
L/R_PS	Posterior superior iliac spine
L/R_TH1-4 Cluster	Cluster of four markers placed on the lateral surface of the thigh
L/R_FLE	Lateral epicondyle
L/R_FME	Medial epicondyle
L/R_SK1-4 Cluster	Cluster of four markers placed on the lateral surface of the shank
L/R_FAL	Lateral prominence of the lateral malleolus
L/R_TAM	Medial prominence of the medial malleolus
L/R_FCC	Aspect of the Achilles tendon insertion on the calcaneus
L/R_FM1	Dorsal margin of the first metatarsal head
L/R_FM5	Dorsal margin of the fifth metatarsal head

#### 2.2.3 Surface electromyography (sEMG) parameters

During the movement, muscles’ sEMG data were recorded using 16 wireless sEMG system (Trigno Wireless EMG System, Delsys Inc., Natick, MA, United States) sampled at a frequency of 2,000 Hz. The muscles under examination are listed in Table 2. Skin preparation and electrodes were placed according to the recommendations of Surface ElectroMyoGraphy for the Non-Invasive Assessment of Muscles guidelines (Hermens et al., 2000). Before the experiment, it is necessary to remove hair from the area to be monitored and gently exfoliate the skin using a damp cloth to eliminate the dead skin layer. This process aids in reducing the impedance between the electrodes and skin, thereby enhancing signal conduction. Subsequently, alcohol-free disinfectant wipes or cotton balls should be used to gently cleanse the skin and eliminate any lingering cleansers, dead skin, or dirt. It is crucial to ensure that the skin is completely dry before securing the sensors (Hermens et al., 2000).

**TABLE 2:** The tested muscles and their abbreviations.

Abbreviations	Muscles’ name
GMed	Gluteus medius
GM	Gluteus maximus
BF	Biceps femoris
ST	Semimembranosus
LG	Lateral gastrocnemius
MG	Medial gastrocnemius
VL	Vastus Lateralis
RF	Rectus femoris
VM	Vastus Medialis
TA	Tibialis anterior

sEMG data processing includes high-pass filtering (400 HZ), rectifying, and low-pass filtering (20 HZ). The normalization of muscles followed the established gold standard, the maximum voluntary isometric contraction test (Hubley-Kozey et al., 2006). During each muscle normalization test, participants followed visual and verbal stimuli. Each muscle slowly began to gain strength, reached maximum effort, held for 3 s, and quickly relaxed (Fu et al., 2021). The normalization test was repeated three times for each muscle with a 60-s rest period in between each test.

### 2.3 Data analysis

All data were collected using Vicon and processed using Visual 3D (V6, C-motion Inc., Germantown, MD, United States) and SPSS 26.0. All data for both mission phases were normalized to 101 data points, corresponding to each percentage of the mission cycles, i.e., from 0% to 100%. The Shapiro–Wilk test was used to check the normality of the data. If the data did not follow a normal distribution, logarithmic transformation was conducted prior to further analysis, such as for STS time and sEMG data. The unpaired Student’s *t*-test was used to compare demographic characteristics and result parameters between groups, with the results reported as mean ± standard deviation $\left(\bar{x}\pm \;s\right)$ . A *p*-value of <0.05 was considered significant.

## 3 Results

In Table 3, the two groups had no significant differences, except for BMI, in demographic data.

**TABLE 3:** Descriptive participant demographics ( $\bar{x}\pm \;s$ ).

	KOA group (*n* = 24)	Control group (*n* = 12)	*p*-value
Age (years)	63.63 ± 4.30	60.92 ± 3.53	0.07
Height (cm)	160.49 ± 7.99	164.08 ± 7.21	0.20
Body mass (kg)	61.67 ± 6.85	56.82 ± 7.50	0.06
BMI (kg/m^2^)^a^	23.93 ± 1.94	21.01 ± 1.31	0.00
K-L grade Ⅰ/grade Ⅱ	5/19	\	\

^a^ means *p* < 0.05.

### 3.1 Temporal and kinematic parameters

Table 4 shows no significant difference in task completion time and the appearance of T1 between the groups. Compared with participants in the control group, patients with KOA had a wider range of pelvic motion in the sagittal plane, a smaller range of motion of the knee and ankle joints in the sagittal plane, and a greater range of motion of the knee and ankle joints in the coronal plane. Additionally, patients with KOA had a lower hip joint maximum angular velocity in the sagittal plane compared to participants in the control group.

**TABLE 4:** Temporal and ROM parameters between KOA and control group ( $\bar{x}\pm \;s$ ).

	KOA group (*n* = 24)	Control group (*n* = 12)	*p*-value
Temporal parameters			
Stand to Sit time (s)^b^	1.56 ± 0.44	1.56 ± 0.28	0.54
T1 time (%)	64.26 ± 5.68	63.43 ± 7.24	0.29
ROM (°) in Sagittal plane			
Pelvic^a^	3.43 ± 1.91	2.93 ± 1.30	0.02
Hip^a^	73.77 ± 12.21	78.32 ± 10.23	0.00
Knee^a^	82.06 ± 7.37	85.96 ± 8.62	0.00
Ankle	17.63 ± 5.72	18.33 ± 4.43	0.21
ROM (°) in coronal plane			
Pelvic	31.76 ± 7.26	31.76 ± 8.15	0.99
Hip	6.27 ± 2.74	6.21 ± 2.52	0.85
Knee^a^	8.09 ± 3.05	7.25 ± 4.03	0.03
Ankle^a^	5.28 ± 2.70	4.07 ± 2.10	0.00
ROM (°) in horizontal plane			
Pelvic	3.75 ± 2.07	4.09 ± 1.30	0.06
Hip	11.87 ± 3.27	10.79 ± 6.00	0.08
Knee	8.40 ± 3.49	8.67 ± 3.65	0.51
Ankle	4.82 ± 1.51	4.89 ± 1.42	0.69
Max velocity (deg/s) in Sagittal plane			
Hip^a^	156.54 ± 31.95	166.48 ± 23.71	0.00
Knee	9.09 ± 7.23	10.52 ± 8.32	0.12
Ankle	39.87 ± 14.11	40.85 ± 13.84	0.55

^a^ means *p* < 0.05.

b means the original data has already been logarithmically transformed.

T1 time (%): The percentage of T1 occurrence time during the STS, task.

### 3.2 Kinetic parameters

**FIGURE 3:**
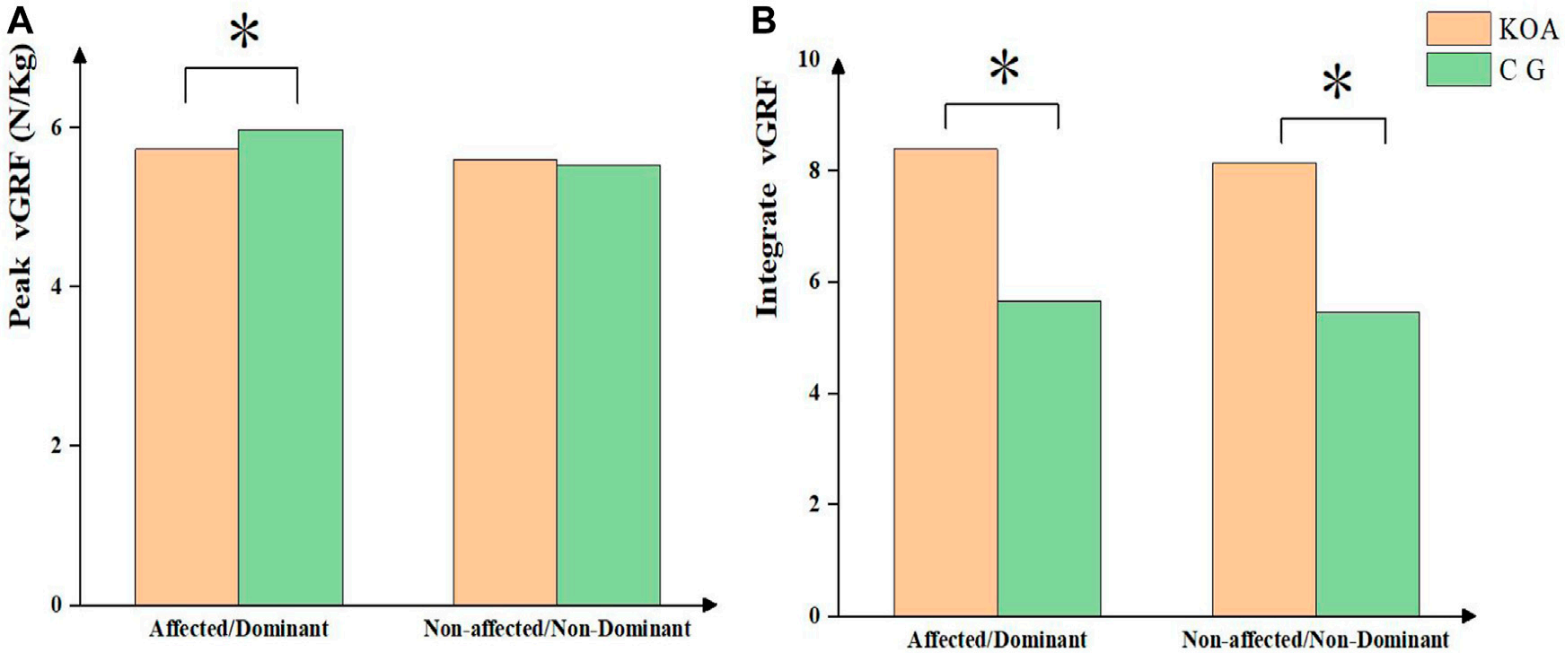
vGRF parameters between KOA and control group.

In Figure 3, patients with KOA had a lower vertical GRF (vGRF) magnitude on the affected side than participants in the control group. However, patients with KOA exhibited a higher integrated GRF on both sides.

**FIGURE 4:**
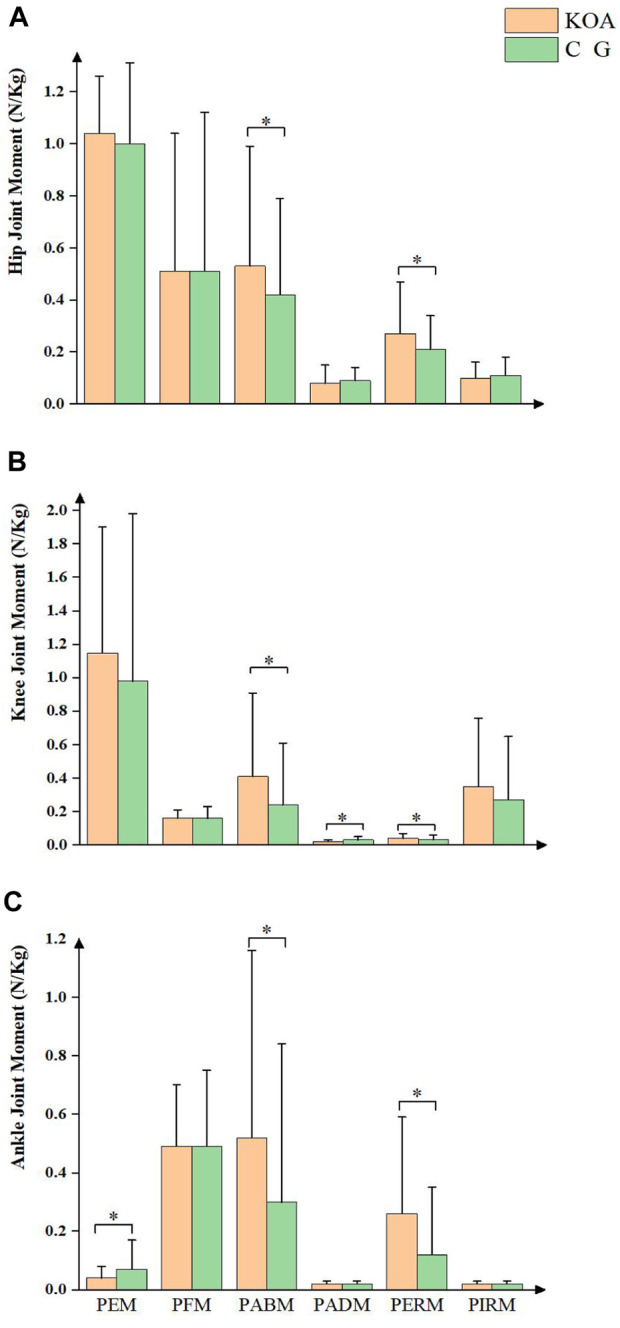
Joint moment between KOA and control group.

The moments of the lower limb joints are shown in Figure 4 during the STS process. Compared with the control group, subjects with KOA demonstrated larger hip PABM and PERM, knee PABM and PERM, as well as ankle PABM and PERM. Furthermore, patients with KOA had smaller knee PADM and ankle PEM.

### 3.3 Surface EMG data

As shown in Table 5, patients with KOA had reduced maximum activation levels of the bilateral gluteus medius (GMed), affected-side gluteus medius (GM), semitendinosus (ST), rectus femoris (RF), and tibialis anterior (TA) muscles compared with the control group; however, the activation level of the biceps femoris (BF) muscle was higher in patients with KOA. Additionally, the integrated electromyography (EMG) values of the affected-side GMed, GM, ST, vastus lateralis (VL), RF, vastus medialis (VM), and TA muscles were lower in patients with KOA; however, the BF muscle showed the opposite trend.

**TABLE 5:** Muscle activation between KOA and control group ( $\bar{x}\pm \;s$ ).

		Peak RMS (%) of muscles			iEMG of muscles		
		KOA	CG	*p*	KOA	CG	*p*
GMed^b^	affected/Dominant	27.95 ± 19.43	49.98 ± 22.26	0.00^a^	21.43 ± 17.61	35.21 ± 18.63	0.00^a^
	Non-affected/Non-Dominant	31.06 ± 20.01	38.35 ± 21.60	0.01^a^	23.59 ± 23.22	25.95 ± 18.91	0.81
GM^b^	affected/Dominant	17.29 ± 8.28	29.09 ± 17.97	0.00^a^	10.78 ± 5.67	17.24 ± 9.49	0.00^a^
	Non-affected/Non-Dominant	22.84 ± 14.81	21.82 ± 13.64	0.90	13.74 ± 12.63	12.70 ± 5.79	0.51
BF^b^	affected/Dominant	19.54 ± 16.30	17.40 ± 18.31	0.00^a^	9.92 ± 9.74	9.56 ± 5.30	0.01^a^
ST^b^	affected/Dominant	19.66 ± 15.59	24.49 ± 23.28	0.00^a^	7.99 ± 10.04	10.98 ± 6.99	0.00^a^
LG^b^	affected/Dominant	18.72 ± 10.76	20.10 ± 15.41	0.82	10.74 ± 6.61	12.11 ± 10.35	0.62
MG^b^	affected/Dominant	19.03 ± 10.47	18.91 ± 11.71	0.46	10.65 ± 5.38	10.59 ± 5.78	0.46
VL^b^	affected/Dominant	64.40 ± 26.42	63.02 ± 25.04	0.66	40.96 ± 24.48	50.70 ± 25.97	0.00^a^
RF^b^	affected/Dominant	57.96 ± 29.93	64.59 ± 16.64	0.00^a^	35.51 ± 19.49	42.42 ± 25.45	0.00^a^
VM^b^	affected/Dominant	50.07 ± 26.88	49.85 ± 23.99	0.35	27.34 ± 13.90	33.97 ± 19.58	0.00^a^
TA^b^	affected/Dominant	22.17 ± 15.31	29.39 ± 14.72	0.00^a^	13.82 ± 8.49	15.31 ± 6.26	0.00^a^

^a^ means *p* < 0.05.

^b^ means that the original data has already been logarithmically transformed.

## 4 Discussion

The biomechanical mechanism analysis of the early identification of KOA could promote the development of health management strategies and improve the predictability of this chronic degenerative disease. STS is a common daily activity essential for upright mobility and can significantly impact the functional mobility and quality of life of patients with KOA. This cross-sectional case-control study aimed to investigate the differences in three-dimensional spatiotemporal, kinematic, and dynamic parameters as well as surface electromyography data between patients with KOA and controls during STS.

Sagittal plane parameters in this study were consistent with those reported in previous studies; however, some differences were noted in the joint ROM, PABM, PERM, and vGRF parameters in the coronal and transverse planes.

The above phenomena may be attributed to the abnormal weight-bearing and adductor muscle recruitment patterns in the lower limbs of patients with early-stage KOA. This highlights the potential of 3D motion screening in the identification and management of early-stage KOA.

This study indicates that subjects with KOA have increased BMI, which is in accordance with results reported in previous studies, suggesting that obesity is a significant factor in KOA (Glyn-Jones et al., 2015). The changes observed in the sagittal plane in this study agree with previous studies (Wu et al., 2015; Bouchouras et al., 2020; Fu et al., 2021). The parameters in the coronal and transverse planes were different between the KOA and control groups.

Previous research has shown that patients with KOA exhibit greater postural sway and longer durations during the STS task than healthy individuals (Wu et al., 2015; Fu et al., 2021), which is considered an indicator of functional mobility deficit (Pollock et al., 2014; Boukadida et al., 2015). It is also associated with a higher fall risk (Cheng et al., 1998; Najafi et al., 2002). However, the temporal parameters observed in this study are consistent with those reported by Bouchouras et al. (2020), who found no statistically significant difference in the task duration between patients with knee osteoarthritis and healthy controls (Bouchouras et al., 2020). This may be because the participants in our study were all patients with early-stage KOA whose symptoms were not yet apparent.

In the sagittal plane, patients with KOA had a smaller ROM of the hip and knee joint, a larger ROM of the pelvis, and a smaller ankle PEM than the healthy individuals. In addition, patients with KOA showed reduced hip and knee joint mobility and lower maximum flexion angular velocity of the hip joint. Similar observations were made previous studies, which found that patients with KOA had an increased anterior pelvic tilt angle (Fu et al., 2021) and decreased knee joint ROM (Wu et al., 2015; Bouchouras et al., 2020).

Trunk anteflexion and ankle dorsiflexion are crucial factors that control the backward and downward displacements of the center of mass of the body during STS maneuvers (Nakagawa and Petersen, 2018). During STS, the pelvic movement of patients with KOA in the sagittal plane was significantly increased, possibly due to the trunk’s increased forward tilt. The forward tilt angle of the trunk was significantly increased to allow the body’s center of gravity to fall within the support surface to maintain stability, perhaps as a strategy to mitigate pain in individuals with advanced KOA (Turcot et al., 2012).

Ankle joint dorsiflexion was reduced in patients with KOA. Prior investigations have reported a reduced ankle dorsiflexion angle in women with KOA during the execution of STS tasks (van Melick et al., 2017; Fu et al., 2021). This alteration in ankle movement during weight-bearing activities may affect dynamic balance. According to a previous report, a decreased range of motion in ankle dorsiflexion may be linked to instability in the anterior–posterior direction, potentially compromising the body’s capacity to lower its center of mass (Nakagawa and Petersen, 2018). Furthermore, limitations in ankle dorsiflexion may result in abnormal knee alignment, leading to an increased risk of progression of KOA (Nakagawa and Petersen, 2018).

The results showed that patients with KOA have increased knee and ankle joint ROM and PABM in lower limb joints in the coronal plane, suggesting a potential decrease in stability among patients with KOA. This could be attributed to the impaired activation of lower limb muscles and excessive activation of the BF muscle. Furthermore, MacKinnon and Winter have shown that hip abductor muscles play a crucial role in load-bearing and balance control during walking (MacKinnon and Winter, 1993). The impaired activation of the hip abductor muscles observed in this study is consistent with their findings. We speculate that muscle activation impairments may increase joint loading, accelerating KOA progression.

Additionally, we found that during the task, patients with KOA had higher values of integrated vGRF on both sides than the control group, and the vGRF magnitude of the lower limb on the affected side was lower in patients with KOA. Previous studies have shown that during STS task, there is an asymmetrical weight transfer in the coronal plane, resulting in the COP moving toward the non-affected side (Cheng et al., 1998; Yamada and Demura, 2009). However, most patients with KOA had mild symptoms in this study, and the load transfer was insignificant. We speculate that as the disease progresses, load bearing in patients with KOA may gradually shift to the unaffected side.

Changes in joint ROM were insignificant between the two groups in the horizontal plane. However, patients with KOA had a greater PERM in lower limb joints than the control group. This may be due to abnormal activation of the BF muscle (Bouchouras et al., 2020). Furthermore, the knee joint had a more significant PIRM than other joints, which may indicate why it is particularly susceptible to KOA (Neogi, 2013). The differences in parameters on the horizontal plane were relatively small; however, as the values were also small in this plane, these subtle differences may still significantly impact KOA progression.

During the task, there are alternating patterns of lower extremity muscle activity (Wu et al., 2015; Bouchouras et al., 2020). Eccentric contractions of the extensor muscles of the knee and hip joints are crucial for reducing movement speed and achieving stable and safe landing on the chair (Ferrante et al., 2005). We found smaller RMS values of GMed, GM, ST, RF, and TA muscles as well as smaller iEMG values of GMed, GM, ST, VL, VM, RF, and TA muscles in the KOA group than in the control group. Meanwhile, the BF muscle in the KOA group exhibited larger RMS and iEMG values than those in the control group. These findings are similar to those reported in the study conducted by Fu and Bouchouras that reported lower activation levels of the quadriceps femoris and other muscles coupled with higher BF muscle activation levels during STS task (Bouchouras et al., 2020; Fu et al., 2021).

An increase in BF muscle activity during daily activities is commonly observed in individuals with KOA (Bouchouras et al., 2020; Fu et al., 2021). This may be attributed to the fact that the BF and RF are bi-articular muscles, and the increased anterior tilt of the trunk increased the activity of the BF muscle. In contrast, the opposite is true for the RF muscle. Another possibility is that BF muscle activation provides additional force to balance and stabilize the knee joint (Mills et al., 2013). However, the compensatory strategy of higher BF muscle activation levels may result in increased energetic costs or joint load (Hortobágyi and DeVita, 2000; Patsika et al., 2011). Pai et al. (1994) suggested that during the execution of the STS task, individuals with KOA have a reduced joint torque at the knee when they increase the hip joint load. However, this study found that in patients with early-stage KOA, except for knee patellofemoral joint contact area and pressure distribution, knee joint torques were higher than normal, particularly in PABM and PERM. This suggests that the increased loads may be associated with the onset and progression of degenerative joint diseases (Felson, 2013).

Previous studies have shown that the knee extensor, particularly the quadriceps femoris muscle, is more affected than any other major lower extremity muscle during the STS task (Hughes et al., 1996; Schenkman et al., 1996). This could be attributed to the development of disuse atrophy and reflex inhibition caused by pain. KOA can impair the voluntary activation of the quadriceps femoris muscle (Anan et al., 2016). In the long term, disuse-induced muscle atrophy may decrease the functional stability of the joint it restrains, rendering it more prone to instability (Pai et al., 1994). Muscle activity and joint contact forces provide the primary joint constraints during functional movements (Flaxman et al., 2012; 2017). Severe instability and denervation of the knee joint are associated with an increased risk and accelerated progression of degenerative joint diseases (Lewek et al., 2004; Glyn-Jones et al., 2015). In daily life, reduced range of motion and diminished muscle activity around the knee in individuals with KOA may contribute to muscle atrophy and weakness around the knee. This could be important for the progression of KOA, highlighting the need for proactive muscle-strengthening exercises for individuals with KOA.

The kinematics of the trunk are crucial in maintaining balance during STS transition (Najafi et al., 2002). However, this study only focused on the lower limbs and did not consider factors related to the trunk. Additionally, the sample size was small, and the relationship between disease progression and lower limb biomechanical changes was not compared. Future studies should conduct large-scale experiments and consider factors such as gender and K-L grade to effectively observe the progression patterns of KOA.

## Data Availability

The raw data supporting the conclusion of this article will be made available by the authors, without undue reservation.
